# Composite pheochromocytoma associated with neurofibromatosis type 1

**DOI:** 10.1002/iju5.12603

**Published:** 2023-06-30

**Authors:** Akira Tachibana, Kota Iida, Yoshitaka Itami, Masaya Hashimura, Yukinari Hosokawa, Kiyohide Fujimoto

**Affiliations:** ^1^ Department of Urology Tane General Hospital Osaka Japan; ^2^ Department of Urology Nara Medical University Nara Japan

**Keywords:** adrenal tumor, composite pheochromocytoma, ganglioneuroma, neurofibromatosis type 1

## Abstract

**Introduction:**

Composite pheochromocytoma is a rare tumor, occurring in only 3% of pheochromocytomas. We report a case of composite pheochromocytoma with neurofibromatosis type 1.

**Case presentation:**

A 42‐year‐old man was referred to our department for further evaluation of an incidentally detected right adrenal tumor. He was a patient at another hospital for neurofibromatosis type 1. The serum and urinary catecholamine levels exceeded the normal range. Abdominal computed tomography and magnetic resonance imaging showed a 2.8 cm diameter right adrenal tumor, and ^123^I‐metaiodobenzyguanidine scintigraphy showed radioisotope uptake. He was diagnosed with pheochromocytoma and underwent a right laparoscopic adrenalectomy. Histopathological examination revealed that the tumor consisted of a pheochromocytoma and ganglioneuroma. The final diagnosis was composite pheochromocytoma‐ganglioneuroma. Five years after surgery, no recurrence was observed.

**Conclusion:**

Preoperative diagnosis of composite pheochromocytoma‐ganglioneuroma is difficult; therefore, histopathological examination is necessary for a definitive diagnosis. Pheochromocytoma management requires lifelong follow‐up.

**Abbreviations & Acronyms**
^123^I‐MIBG: ^123^I‐metaiodobenzylguanidine
CT: computed tomography
H&E: hematoxylin and eosin
MEN: multiple endocrine neoplasia
MRI: magnetic resonance imaging
NF1: neurofibromatosis type 1
VHL: von Hippel–Lindau

**Keynote message**

We report on a case of composite pheochromocytoma associated with neurofibromatosis type 1. Adrenalectomy is the gold standard treatment for composite pheochromocytoma, and lifelong follow‐up is required.

## Introduction

Pheochromocytomas were once termed “the 10% tumor”; that is, 10% of all pheochromocytomas were considered malignant and hereditary.^1^, ^2^ However, all pheochromocytomas and paragangliomas are considered to have metastatic potential and are categorized as malignant according to the latest World Health Organization classification.^3^ Around 30%–40% of patients with pheochromocytomas have identifiable germline mutations, including VHL syndrome, NF1, and MEN syndrome type II.^4^ The Endocrine Society guidelines suggest genetic testing for all patients with pheochromocytomas and paragangliomas.^5^

Composite pheochromocytoma is a rare tumor histologically composed of pheochromocytoma and other neurogenic tumor components.^6^ This case report describes a patient with NF1 who underwent adrenalectomy and was diagnosed with composite pheochromocytoma‐ganglioneuroma.

## Case presentation

**Fig. 1:**
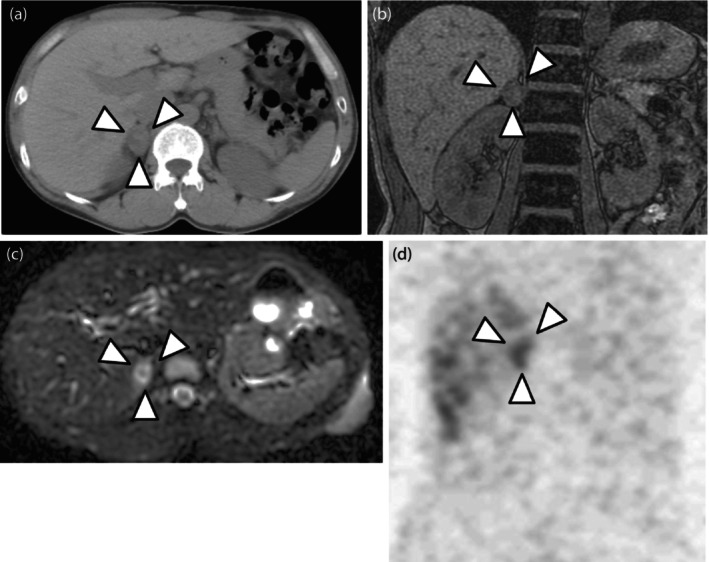
(a) CT shows a right adrenal tumor that is 2.8 cm in diameter. (b) MRI shows a hypointense mass on T1 weighted images in the right adrenal gland. (c) Diffusion‐weighted images show a heterogeneous tumor in the same region. (d) ^123^I‐MIBG scintigraphy confirming a localized increased uptake in the same region.

**Fig. 2:**
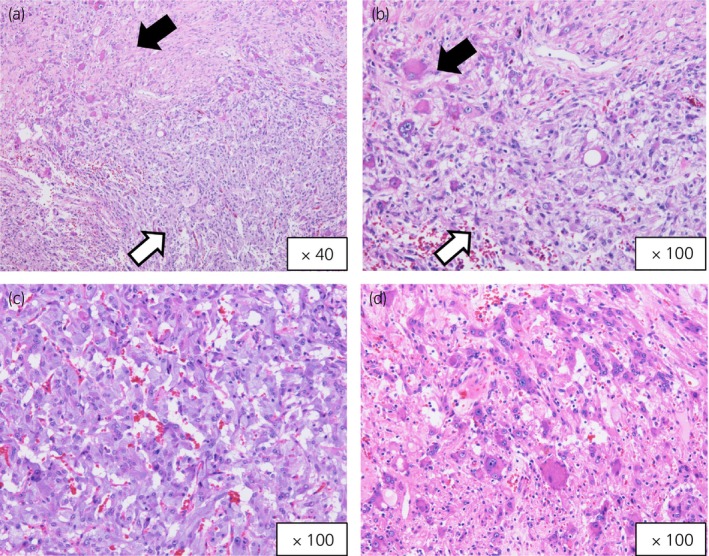
(a, b) Two different components are observed in the tumor (H&E stain): pheochromocytoma (white arrow) and ganglioneuroma (black arrow). (c) Pheochromocytoma component of the composite tumor (H&E). (d) Ganglioneuroma component consisting of ganglion and Schwann cells (H&E stain).

**Fig. 3:**
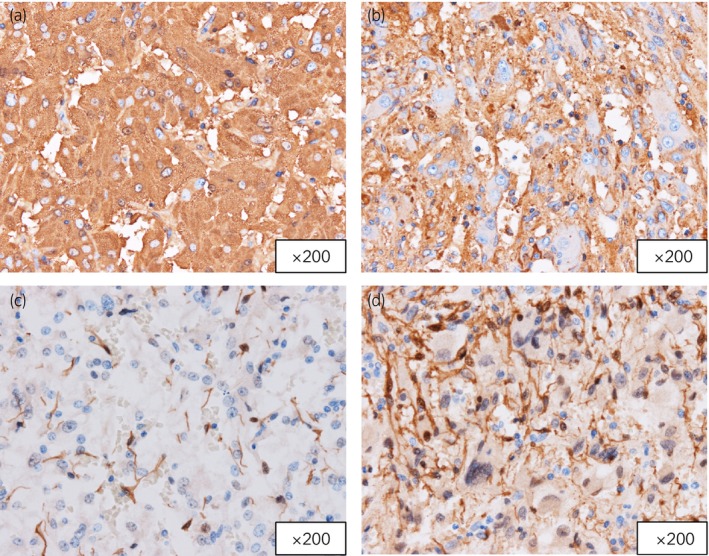
(a) The chromaffin cells in pheochromocytoma are strongly immunoreactive for chromogranin A. (b) The ganglion cells are negative or weakly immunoreactive for chromogranin A. (c) The chromaffin cells are negative immunoreactive for S100. (d) Schwann cells are immunoreactive for S100.

A 42‐year‐old man was referred to our department for further evaluation of an incidentally detected right adrenal tumor. He was a patient at another hospital for NF1 and had a family history; his father and son had NF1 as well. He had no symptoms, such as sweating, palpitation, or headache, and had no history of hypertension. Abdominal examination revealed café au lait spots and many neurofibromas. Blood examination revealed mild increases in adrenaline (0.18 ng/mL; normal range < 0.1) and noradrenaline (1.01 ng/mL; normal range 0.20–0.50). Urine analysis revealed high levels of adrenaline (76.9 μg/day; normal range 3–41), noradrenaline (271.2 μg/day; normal range 31–160), and dopamine (1941.1 μg/day; normal range 280–1100). CT revealed a right adrenal tumor that was 2.8 cm in diameter (Fig. 1a). MRI showed a hypointense mass on T1 weighted images and a heterogeneous tumor on diffusion‐weighted images of the right adrenal gland (Fig. 1b,c). ^123^I‐MIBG scintigraphy confirmed a localized increase in uptake in the same region (Fig. 1d). We suspected pheochromocytoma and performed right laparoscopic adrenalectomy after alpha blocker therapy. Histopathological examination revealed a composite tumor with two different components (Fig. 2a,b). One component was pheochromocytoma (Fig. 2c) and the other was ganglioneuroma, which consisted of ganglion and Schwann cells (Fig. 2d). Chromaffin cells in pheochromocytomas were strongly immunoreactive for chromogranin A (Fig. 3a). The ganglion cells were either negative or weakly immunoreactive for chromogranin A (Fig. 3b). Chromaffin cells were negative for S100 (Fig. 3c), and Schwann cells were immunoreactive for S100 (Fig. 3d). The patient was diagnosed with a composite pheochromocytoma‐ganglioneuroma, underwent follow‐up with biochemical evaluation and abdominal CT or MRI once a year, and had no recurrence or metastases 5 years postoperatively.

## Discussion

Composite pheochromocytoma is a rare tumor that occurs in only 3% of pheochromocytoma.^7^ The tumor types that co‐exist with pheochromocytoma are ganglioneuromas, ganglioneuroblastomas, neuroblastomas, peripheral nerve sheath tumors, and neuroendocrine carcinomas.^8^ Embryologically, both pheochromocytomas and neurogenic tumors are derived from the neural crest cells.^7^ The diagnosis of a composite pheochromocytoma is solely based on histopathological findings.^1^ A systematic review of 94 patients with composite pheochromocytomas showed that the incidence rate of composite pheochromocytoma‐ganglioneuroma was 65%, the most frequently occurring composite pheochromocytoma.^9^ Shawa *et al*. reported difficulty in distinguishing composite pheochromocytoma‐ganglioneuroma from pure pheochromocytoma clinically and radiologically and suggested they should be managed similarly.^10^

Ganglioneuromas are rare benign tumors composed of mature ganglion and Schwann cells.^11^ They commonly occur in the posterior mediastinum and retroperitoneum, but rarely in the adrenal gland.^12^ Adrenal ganglioneuromas have been reported to be associated with genetic syndromes, such as NF1, neurofibromatosis type 2, and MEN 2.^13^ Patients with adrenal ganglioneuromas are largely asymptomatic; however, they have been reported to elevate catecholamine levels (in approximately 30% of cases).^12^ Qing *et al*. reported that the proper diagnosis rate of adrenal ganglioneuromas using CT or MRI was 35.3%.^14^ Because the preoperative diagnosis of adrenal ganglioneuroma is difficult, histopathological examination is required for a definitive diagnosis.^12^ Although no cases of recurrence or malignant transformation have been reported in adrenal ganglioneuroma, Kulkarni *et al*. reported malignant transformation of ganglioneuroma in the spinal cord.^15^ In the present case, we suspected pheochromocytoma on the basis of biological and radiological findings, but it was difficult to differentiate the component of ganglioneuroma on preoperative diagnostic imaging.

Composite pheochromocytoma is often associated with genetic syndromes, such as NF1, MEN 2, VHL syndrome, and watery‐diarrhea hypokalemia‐achlorhydria syndrome.^9^ It has been reported that 19% of the composite pheochromocytomas are associated with NF1.^9^ *NF1* gene encodes a large protein of 2818 amino acids, called neurofibromin.^16^ Kimura *et al*. reported that loss of neurofibromin in patients with NF1 may induce abnormal proliferation of Schwann cells in composite pheochromocytomas as well as in neurofibromatosis.^17^ In patients with NF1‐related pheochromocytoma, Nolting *et al*. suggested lifelong follow‐up with clinical and biochemical evaluation once a year and abdominal MRI every 5 years because the rate of asymptomatic patients is high.^18^ The rate of asymptomatic patients with NF1‐related pheochromocytoma is more than 80%.^19^ In the present case, the patient had a history of NF1 and had a right adrenal tumor, and he was asymptomatic. For further follow‐up, we should explain the possibility of recurrence to patients with NF1‐related pheochromocytoma even if the patients are asymptomatic. As the recurrence rate of composite pheochromocytoma‐ganglioneuroma is unclear, the patient was followed up according to pheochromocytoma management, and no recurrence was observed. The clinical behavior of composite pheochromocytoma is associated with neural components, such as ganglioneuroblastomas, neuroblastomas, and malignant peripheral nerve sheath tumors.^20^ It has been reported that composite pheochromocytoma‐ganglioneuroma have the same prognosis as pure pheochromocytoma.^20^

In conclusion, we described a case of composite pheochromocytoma‐ganglioneuroma associated with NF1. The treatment outcome of composite pheochromocytoma‐ganglioneuroma is similar to that of pure pheochromocytoma, and lifelong follow‐up is required according to the same management.

## Author contributions

Akira Tachibana: Writing – original draft. Kota Iida: Writing – review and editing. Yoshitaka Itami: Resources. Masaya Hashimura: Resources. Yukinari Hosokawa: Supervision. Kiyohide Fujimoto: Supervision.

## Conflict of interest

The authors declare no conflict of interest.

## Approval of the research protocol by an Institutional Reviewer Board

Not applicable.

## Informed consent

We obtained consent from the patient for publication.

## Registry and the Registration No. of the study/trial

Not applicable.
